# Parametric Estimation of Reference Signal Intensity for Semi-Quantification of Tau Deposition: A Flortaucipir and [^18^F]-APN-1607 Study

**DOI:** 10.3389/fnins.2021.598234

**Published:** 2021-06-21

**Authors:** Huiwei Zhang, Min Wang, Jiaying Lu, Weiqi Bao, Ling Li, Jiehui Jiang, Chuantao Zuo, and Alzheimer’s Disease Neuroimaging Initiative

**Affiliations:** Alzheimer’s Association; Alzheimer’s Drug Discovery Foundation; Araclon Biotech; BioClinica, Inc.; Biogen; Bristol- Myers Squibb Company; CereSpir, Inc.; Eisai, Inc.; Elan Pharmaceuticals, Inc.; Eli Lilly and Company; EuroImmun; F. Hoffmann-La Roche Ltd. and its affiliated company Genentech, Inc.; Fujirebio; GE Healthcare; IXICO, Ltd.; Janssen Alzheimer Immunotherapy Research & Development, LLC; Johnson & Johnson Pharmaceutical Research & Development LLC; Lumosity; Lundbeck; Merck & Co., Inc.; Meso Scale Diagnostics, LLC.; NeuroRx Research; Neurotrack Technologies; Novartis Pharmaceuticals Corporation; Pfizer, Inc.; Piramal Imaging; Servier; Takeda Pharmaceutical Company; and Transition Therapeutics.; ^1^PET Center, Huashan Hospital, Fudan University, Shanghai, China; ^2^Shanghai Institute for Advanced Communication and Data Science, Shanghai University, Shanghai, China

**Keywords:** APN-1607, flortaucipir, tau, neurodegeneration, Alzheimer’s disease

## Abstract

**Background:**

Tau positron emission tomography (PET) imaging can reveal the pathophysiology and neurodegeneration that occurs in Alzheimer’s disease (AD) *in vivo*. The standardized uptake value ratio (SUVR) is widely used for semi-quantification of tau deposition but is susceptible to disturbance from the reference region and the partial volume effect (PVE). To overcome this problem, we applied the parametric estimation of reference signal intensity (PERSI) method—which was previously evaluated for flortaucipir imaging—to two tau tracers, flortaucipir and [^18^F]-APN-1607.

**Methods:**

Two cohorts underwent tau PET scanning. Flortaucipir PET imaging data for cohort I (65 healthy controls [HCs], 60 patients with mild cognitive impairment [MCI], and 12 AD patients) were from the AD Neuroimaging Initiative database. [^18^F]-APN-1607 ([^18^F]-PM-PBB3) PET imaging data were for Cohort II, which included 21 patients with a clinical diagnosis of amyloid PET-positive AD and 15 HCs recruited at Huashan Hospital. We used white matter (WM) postprocessed by PERSI (PERSI-WM) as the reference region and compared this with the traditional semi-quantification method that uses the whole cerebellum as the reference. SUVRs were calculated for regions of interest including the frontal, parietal, temporal, and occipital lobes; anterior and posterior cingulate; precuneus; and Braak I/II (entorhinal cortex and hippocampus). Receiver operating characteristic (ROC) curve analysis and effect sizes were used to compare the two methods in terms of ability to discriminate between different clinical groups.

**Results:**

In both cohorts, regional SUVR determined using the PERSI-WM method was superior to using the cerebellum as reference region for measuring tau retention in AD patients (e.g., SUVR of the temporal lobe: flortaucipir, 1.08 ± 0.17 and [^18^F]-APN-1607, 1.57 ± 0.34); and estimates of the effect size and areas under the ROC curve (AUC) indicated that it also increased between-group differences (e.g., AUC of the temporal lobe for HC vs AD: flortaucipir, 0.893 and [^18^F]-APN-1607: 0.949).

**Conclusion:**

The PERSI-WM method significantly improves diagnostic discrimination compared to conventional approach of using the cerebellum as a reference region and can mitigate the PVE; it can thus enhance the efficacy of semi-quantification of multiple tau tracers in PET scanning, making it suitable for large-scale clinical application.

## Introduction

Tau protein is a microtubule-associated protein that is abundantly expressed in the central nervous system (Goedert et al., 1991). Abnormal tau hyperphosphorylation in neurons causes the protein to self-aggregate and form paired helical filaments (PHFs) that contribute to the pathogenesis of neurodegenerative diseases. In Alzheimer’s disease (AD), the deposition of amyloid β (Aβ) is the initial pathologic event leading to the formation of senile plaques (SPs) followed by neurofibrillary tangles (NFTs), leading to neuronal loss and cognitive decline (Braak and Braak, 1991). Diseases involving tau protein misfolding or hyperphosphorylation and accumulation are known as tauopathies; these include AD, frontotemporal dementia with parkinsonism linked to chromosome 17 (FTDP-17), Pick’s disease, progressive supranuclear palsy (PSP), corticobasal degeneration (CBD), and chronic traumatic encephalopathy (Villemagne et al., 2015; Okamura et al., 2018). According to the 2018 National Institute on Aging and Alzheimer’s Association guidelines for AD, SPs and NFTs can be detected *in vivo* by positron emission tomography (PET) imaging with Aβ and tau tracers to differentiate dementia from other neurodegenerative diseases. In addition to Aβ deposition, tau retention is a major factor contributing to AD pathogenesis.

The first generation of radiolabeled tau tracers for PET were developed based on different binding targets of tau PHFs such as quinoline derivatives (e.g., [^18^F]THK523, [^18^F]THK5105, [^18^F]THK5117, and [^18^F]THK5351), PBB3-based tracers (e.g., [^11^C]PBB3), and benzimidazole pyrimidine derivatives (e.g., flortaucipir/[^18^F]-AV-1451/[^18^F]-T807 and [^18^F]T808). The second generation of tracers show improved binding selectivity and pharmacokinetics and include [^18^F]AM-PBB3 and [^18^F]-APN-1607/[^18^F]PM-PBB3 (Leuzy et al., 2019); and [^18^F]RO-948, [^18^F]GTP1, and [^18^F]MK-6240 (Okamura et al., 2018). In AD clinical trial, these tracers have demonstrated high binding affinity for tau PHFs and tau selectivity (Scholl et al., 2016; Shimada et al., 2017; Sone et al., 2017; Lu et al., 2020). Additionally, the amount of tracer that was retained and the brain regions in which it was detected in AD was significantly correlated with the clinical severity of dementia (Devous et al., 2018; Villemagne et al., 2018). This is one of the advantages of tau PET over Aβ PET, as amyloid burden shows little association with dementia severity (Rabinovici and Jagust, 2009). Reliable and reproducible methods for tau quantification in PET images are critical for diagnosis.

Semi-quantitative methods are non-invasive and widely used to eliminate the effects of arterial blood sampling in clinical studies, and have been applied to tau PET in AD (Shcherbinin et al., 2016). Conventionally, in PET images of target-specific biomarker retention, AD biomarkers are measured by standardized uptake value ratio (SUVR) in regions of interest (ROIs), which is the ratio of average activity concentration in the target ROI relative to that in the reference region. In the calculation of SUVR, inter-subject variation should be minimized. Global mean normalization or normalization to an anatomic reference region can be done easily in clinical practice as it does not require complex mathematical modeling. Proper normalization reference region can enhance cross-sectional accuracy and longitudinal coherence in PET studies (Zhang et al., 2017). Optimal reference regions vary for different PET tracers, and include the cerebellum, pons, and sensorimotor cortex.

The cerebellum was shown to be devoid of NFT deposition (Marquie et al., 2015); therefore, the whole cerebellum or cerebellar gray matter (GM) is commonly used as reference region for tau PET in AD. In several studies, tau PET images were resampled by normalizing the subject-specific magnetic resonance imaging (MRI) template to the T1 MRI template for spatial normalization of ROIs, with the cerebellar GM as the reference (Shcherbinin et al., 2016; Kang et al., 2017; Kitamura et al., 2018; Wong et al., 2018; Lohith et al., 2019; Lu et al., 2020; Mueller et al., 2020). However, cerebellar GM or the whole cerebellum has the disadvantages of small size, low signal detection sensitivity, and susceptibility to noise and truncation. In particular, in longitudinal studies, the partial volume effect (PVE) can cause a spillover or cross-contamination of counts between adjacent structures due to limited spatial resolution (Meechai et al., 2015). Therefore, a reliable technique for tau PET image analysis is needed that minimizes the influence of these factors.

A subject-specific, data-driven technique known as parametric estimation of reference signal intensity (PERSI) was recently proposed for the analysis of flortaucipir PET images (Southekal et al., 2018). This method reduces inter-subject variability while enhancing discrimination between cohorts by using white matter (WM) as the reference region for count normalization based on signal intensity histograms, as tau binding by WM is considered negligible. PERSI can mitigate the PVE by distinguishing voxels associated with non-specific binding, which have lower signal intensity, from those that reflect contamination.

To determine whether the PERSI method is applicable to different tau tracers, in this study we applied the method to the analysis of data from tau PET imaging with flortaucipir and [^18^F]-APN-1607. We first retested the PERSI method in a cohort that underwent PET imaging with flortaucipir as the tau tracer (Cohort I, including healthy controls [HCs] and patients with mild cognitive impairment [MCI] and AD), which has been previously reported. As PERSI has not been validated for second-generation tau tracers, we then applied the method to a second cohort that underwent PET imaging with [^18^F]-APN-1607 (Cohort II, including HCs and AD patients). We evaluated the count normalization performance of PERSI using WM as a reference region in the PET images.

## Materials and Methods

### Subjects

We retrospectively analyzed data from two cohorts who underwent tau PET scans. Flortaucipir PET scans and corresponding structural MRI scans for Cohort I was obtained from the AD Neuroimaging Initiative (ADNI) database^1^ and its extensions. This cohort consisted of 65 HCs along with 60 MCI and 12 AD patients. The primary goal of the ADNI was to test whether serial MRI and PET imaging findings, biological markers, and data from clinical and neuropsychologic assessments can be combined to predict and measure the progression of MCI and AD. In Cohort II, 21 AD patients clinically diagnosed as amyloid PET-positive (based on visual evaluation by three nuclear medicine specialists with 8 years of clinical experience on average) and 15 HCs underwent [^18^F]-APN-1607 PET scanning. Clinically probable AD was determined based on current diagnostic criteria (McKhann et al., 2011). Experienced neurologists from the cognitive impairment clinic administered the Mini-Mental State Examination (MMSE) to all subjects. The demographic and clinical characteristics of the participants are summarized in Table 1.

**TABLE 1:** Clinical and demographic characteristics of the study subjects.

Group		Sex, M/F	Age, years	Education, years	MMSE	MoCA	CDR-SB
Flortaucipir (ADNI database)	HC (65)	24/41	74.1 ± 3.89	16.6 ± 2.32	29.2 ± 0.98	26.4 ± 2.47	0.046 ± 0.14
	MCI (60)	21/39	75.0 ± 5.96	16.3 ± 2.68	28.3 ± 1.74*	24.8 ± 2.76	1.18 ± 0.75*
	AD (12)	6/6	77.5 ± 9.71	16.4 ± 2.57	22.7 ± 2.42*	18.1 ± 4.36	5 ± 2*
[^18^F]-APN-1607	HC (15)	10/5	60.8 ± 4.3	9.47 ± 3.3	27.6 ± 1.3	/	0
	AD (21)	11/10	60.3 ± 10.6	11.3 ± 5.15*	16.7 ± 7.6*	/	8.62 ± 3.8*

Data are presented as mean ± standard deviation.

*P < 0.05 (two-sample t-test).

*AD, Alzheimer’s disease; ADNI, Alzheimer’s Disease Neuroimaging Initiative; CDR-SB, Clinical Dementia Rating – Sum of Boxes; HC, healthy control subjects; MCI, patients with mild cognitive impairment; MoCA, Montreal Cognitive Assessment; MMSE, Mini-Mental State Examination.*

The inclusion and exclusion criteria for the diagnostic categories of ADNI were as follows: (1) age between 55–85 years; (2) participants underwent flortaucipir PET and structural MRI scanning; (3) participants underwent a battery of neuropsychologic and cognitive examinations including the Montreal Cognitive Assessment (MoCA), Clinical Dementia Rating–Sum of Boxes (CDR-SB), and MMSE; (4) HCs had MMSE scores between 24–30 (inclusive), were non-depressed, non-MCI, and non-demented; and (5) severe AD patients with MMSE < 10 or MoCA < 10 were excluded. The detailed diagnosis-specific inclusion and exclusion criteria for the HC, MCI, and AD groups can be found in the ADNI dataset^2^.

All procedures in this study were in accordance with the ethical standards of the institutional research committee and with the Helsinki Declaration of 1975 and its later amendments. This study was approved by the institutional review boards of ADNI and the Institutional Review Board of Huashan Hospital (HIRB), Fudan University, China (no. 2018-363). Written, informed consent was obtained from each subject.

### PET Imaging and Data Preprocessing

Flortaucipir PET images for Cohort I were obtained 75–105 min after administration of 370 MBq (10.0 mCi) ± 10% flortaucipir. Detailed information on data acquisition is provided in the study protocol in the ADNI database.

Subjects in Cohort II were scanned with a Siemens Biograph 64 PET/computed tomography (CT) system (Siemens, Erlangen, Germany) in three-dimensional (3D) mode at Huashan Hospital. A low-dose CT transmission scan was performed before PET scanning for attenuation correction. Static emission scans were acquired 90–110 min after intravenous injection of 370 MBq [^18^F]-APN-1607. Image reconstruction was performed with the ordered subset expectation maximization 3D method with six iterations and 21 subsets, Gaussian filtering, and a full width at half-maximum (FWHM) of 3.5 mm. The subjects also underwent anatomic MRI in a 3.0-T horizontal magnet (Discovery MR750; GE Medical Systems, Boston, MA, United States) at Huashan Hospital (Lu et al., 2020).

Positron emission tomography image preprocessing was performed using the Statistical Parametric Mapping 12 (SPM 12; Wellcome Department of Cognitive Neurology, University College London, London, UK) package in Matlab (MathWorks, Sherborn, MA, United States). The unified segmentation and normalization algorithms of SPM12 were used to spatially normalize the T1-weighted MR images acquired at screening to the Montreal Neurological Institute (MNI) brain template while simultaneously generating probabilistic segmentations for GM, WM, and cerebrospinal fluid. The PET images were also spatially normalized to MNI space using deformation field images generated from the MRI segment.

### PET Quantification Analysis

We evaluated the performance of the PERSI method for [^18^F]-APN-1607 PET imaging quantification. The threshold of the WM probabilistic segmentation from individual T1-weighted MR images was set as 0.9 to generate a binary WM image. PET images spatially normalized to MNI space were masked with the individual WM image. The voxels within this WM region were plotted as a histogram and then fitted to a bimodal Gaussian distribution using a non-linear trust region reflective algorithm. For higher intensity peaks, the PERSI method identifies voxels with contamination based on counts from adjacent cortical tissues with confirmed flortaucipir uptake; lower intensity peaks reflect a stable reference signal intensity. We removed voxels in higher intensity peaks and retained those in lower intensity peaks (i.e., the FWHM of the lower peak location) as the individual reference region. Thus, PET images for each subject used this subject-specific WM region as the reference for count normalization and smoothing with a Gaussian filter of 8 mm FWHM (Southekal et al., 2018).

For comparison, we calculated the SUVR of different groups using additional reference regions: (1) the traditional reference region consisting of the whole cerebellum, and (2) WM based on PERSI. The whole brain was parcellated into the following regions for SUVR calculations: frontal, parietal, temporal, and occipital lobes; anterior and posterior cingulate; precuneus; and Braak I/II (entorhinal cortex and hippocampus) (Scholl et al., 2016), which are regions known to be associated with progressive neurodegeneration in AD. The whole cerebellum and all ROIs were manually delineated on the Automated Anatomical Labeling template (Tzourio-Mazoyer et al., 2002).

### Statistical Analysis

Demographic characteristics were compared between AD and HC groups using the two-sample *t*-test or chi-squared test. Effect sizes for the ability of SUVR to discriminate between dementia patients and HC subjects were evaluated with Cohen’s d (*d* = [mean_1_-mean_2_]/sqrt[(std_1_^2^ + std_1_^2^)/2]). We also carried out receiver operating characteristic (ROC) curve analyses for each regional SUVR to assess the capacity for discrimination between diagnostic groups based on the area under the ROC curve (AUC) value. All statistical analyses were performed using SPSS v22.0 software (SPSS Inc., Chicago, IL, United States). *P*-values < 0.05 were considered significant.

## Results

### Application of the PERSI Method to Flortaucipir PET Images

**FIGURE 1:**
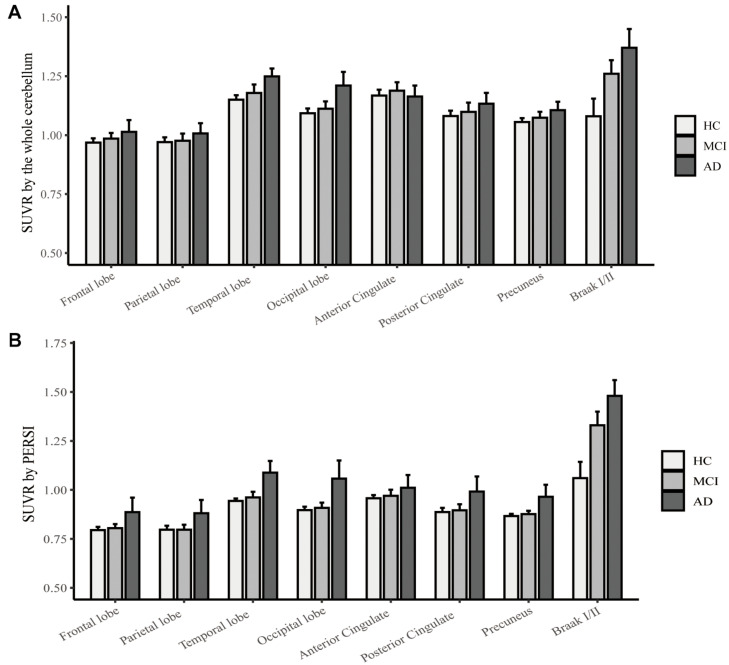
Regional flortaucipir SUVR count normalization. **(A)** The whole cerebellum was the reference region for quantification. **(B)** PERSI-WM for HC, MCI, and AD groups. Mean group SUVRs (±SD) across predefined ROIs are shown.

To compare quantitative efficacy using PERSI-WM vs the whole cerebellum as the reference region, we examined flortaucipir SUVRs within each group of predefined ROIs. SUVRs derived from PERSI-WM revealed significantly higher tau retention in the frontal, parietal, temporal, and occipital lobes, posterior cingulate, precuneus, and Braak I/II in AD and MCI patients compared to HCs (Figure 1). Braak I/II had the highest tau retention (1.51 ± 0.23). Interestingly, in the anterior cingulate, SUVRs derived from the cerebellum showed comparable tau retention in MCI and AD patients, whereas SUVRs derived from PERSI-WM showed higher tau retention in the AD group than in the MCI group, suggesting that this method is superior to using the cerebellum as a reference region.

We next carried out ROC curve analysis and calculated effect sizes to evaluate the diagnostic utility of PERSI-WM compared to the cerebellum (Table 2). PERSI-WM yielded larger effect sizes and AUCs in all ROIs than the cerebellum; the AUCs were 0.517–0.951 between the HC and AD groups and 0.472–0.874 between the HC and MCI groups in all ROIs. PERSI-WM had the largest effect size (Cohen’s d) compared to HCs (AD, 1.37; MCI, 0.282) in the temporal lobe and the largest AUC compared to HCs (AD, 0.951; MCI 0.874) in Braak I/II. Estimates of the effect size and AUCs indicated that PERSI-WM increased between-group differences compared to the whole cerebellum (Table 2).

**TABLE 2:** Mean standardized uptake value ratio of cortical regions by diagnostic group (Cohort I).

Cortical region	Reference area^†^	AUC		Effect size^‡^	
			
		HC-MCI	HC-AD	HC-MCI	HC-AD
Frontal lobe	Cerebellum	0.561	0.517	0.198	0.33
	PERSI-WM	0.535	**0.561**	0.178	**0.70**
Parietal lobe	Cerebellum	0.513	0.521	0.056	0.27
	PERSI-WM	0.508	**0.60**	0.01	**0.64**
Temporal lobe	Cerebellum	0.581	0.753	0.252	0.82
	PERSI-WM	0.523	**0.893**	0.282	**1.37**
Occipital lobe	Cerebellum	0.546	0.697	0.183	0.74
	PERSI-WM	**0.595**	**0.801**	0.161	**1.01**
Anterior cingulate	Cerebellum	0.572	0.642	0.22	0.51
	PERSI-WM	**0.598**	0.615	**0.28**	0.449
Posterior cingulate	Cerebellum	0.503	0.533	0.137	0.35
	PERSI-WM	**0.521**	**0.633**	**0.211**	**0.72**
Precuneus	Cerebellum	0.554	0.597	0.209	0.44
	PERSI-WM	0.472	**0.681**	**0.229**	**0.928**
Braak I/II	Cerebellum	0.762	0.856	0.476	0.672
	PERSI-WM	0.874	**0.951**	0.521	**0.923**

*^†^Cerebellum, the whole cerebellum served as a reference region for quantification; PERSI-WM, parametric estimation of reference signal intensity with white matter as the reference region.*

*^‡^Presented as standardized Cohen’s d; values in bold type represent superior diagnostic ability.*

*AD, Alzheimer’s disease; AUC, area under the receiver operating characteristic curve; HC, healthy control subjects; MCI, patients with mild cognitive impairment; SUVR, standardized uptake value ratio.*

### Validation of the PERSI Method With [^18^F]-APN-1607 PET Images

**FIGURE 2:**
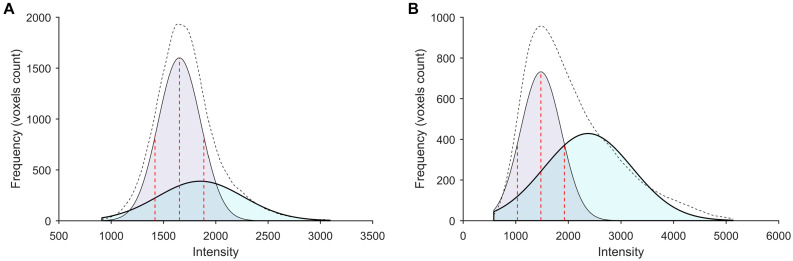
Subject-specific WM histograms for two typical subjects evaluated by [^18^F]-APN-1607 PET. **(A)** A 56-year-old HC (male). **(B)** A 56-year-old AD patient (female). Purple and cyan areas represent lower- and higher-intensity signals, respectively; the black dotted line shows the sum of two peaks (bimodal Gaussian distribution). For AD patients, voxels that spilled into the WM were captured by the higher peak.

Typical signal intensity histograms of the WM used to derive the PERSI reference region for representative participants (HC and AD patient) are shown in Figure 2. In HCs, only a single major peak was observed whereas in AD patients, voxels that had spilled into the WM were captured by a higher peak.

**FIGURE 3:**
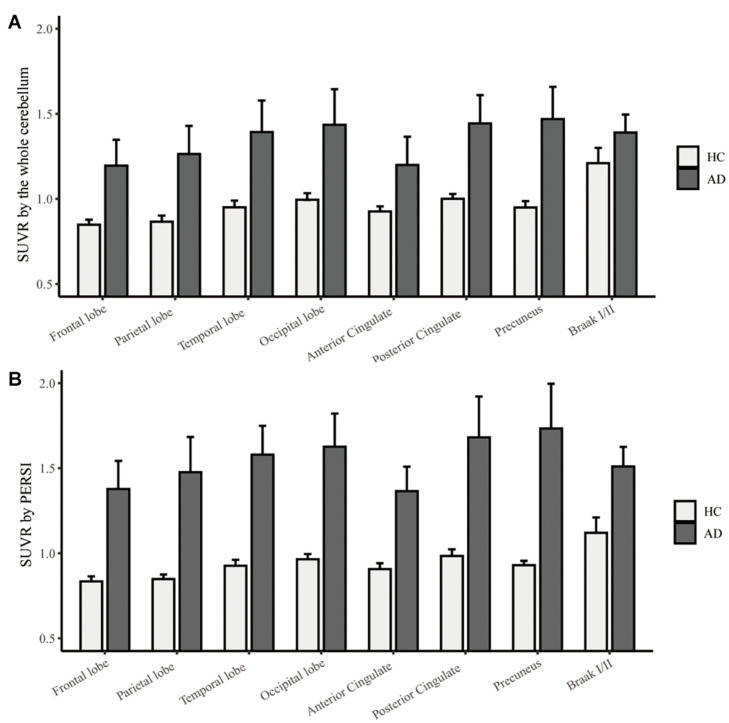
Regional [^18^F]-APN-1607 SUVR count normalization. **(A)** The whole cerebellum was the reference region for quantification. **(B)** PERSI-WM for HC and AD groups. Mean group SUVRs (±SD) across predefined ROIs are shown.

To compare the quantitative efficacy between PERSI-WM and cerebellum as reference regions, we examined [^18^F]-APN-1607 SUVRs within each group of predefined ROIs. SUVRs derived from PERSI-WM showed significantly higher tau retention in the frontal, parietal, temporal, and occipital lobes; anterior and posterior cingulate; precuneus and Braak I/II in the AD group than in the HC group (Figure 3), with the highest tau retention in precuneus (1.73 ± 0.53).

We next carried out ROC curve analysis and calculated effect sizes to evaluate the diagnostic utility of PERSI-WM compared to the cerebellum. PERSI-WM had larger effect sizes and AUCs in ROIs compared to the cerebellum (Table 3). PERSI-WM had the largest effect size (Cohen’s d) compared to HCs in the precuneus (AD: 3.64) and in the temporal lobe (AD: 2.53). The AUC between the HC and AD groups was 0.933–0.975 in all ROIs. Estimates of the effect size and AUCs indicated that the PERSI-WM method increased between-group differences compared to the whole cerebellum (Table 3).

**TABLE 3:** Mean standardized uptake value ratio of cortical regions by diagnostic group (Cohort II).

Cortical region	Reference^†^	AUC	Effect size^‡^
Frontal lobe	Cerebellum	0.914	1.52
	PERSI-WM	0.975	2.38
Parietal lobe	Cerebellum	0.930	1.55
	PERSI-WM	0.956	2.97
Temporal lobe	Cerebellum	0.937	1.61
	PERSI-WM	0.949	2.53
Occipital lobe	Cerebellum	0.922	1.54
	PERSI-WM	0.971	2.66
Anterior cingulate	Cerebellum	0.803	1.15
	PERSI-WM	0.949	1.79
Posterior cingulate	Cerebellum	0.921	1.83
	PERSI-WM	0.933	2.48
Precuneus	Cerebellum	0.943	1.93
	PERSI-WM	0.968	3.64
Braak I/II	Cerebellum	0.823	0.64
	PERSI-WM	0.937	1.33

*^†^Cerebellum, the whole cerebellum served as a reference region for quantification; PERSI-WM, parametric estimation of reference signal intensity with white matter as the reference region.*

*^‡^Presented as standardized Cohen’s d.*

*AUC, area under the receiver operating characteristic curve.*

## Discussion

In tauopathies, tau PHFs can be non-invasively detected *in vivo* by PET imaging with different tau tracers such as benzimidazole pyrimidine derivatives (flortaucipir) and PBB3-based tracers ([^18^F]-APN-1607). In this study, we evaluated the utility and reliability of the PERSI method for analyzing data from PET imaging using 2 different tau tracers—i.e., flortaucipir and [^18^F]-APN-1607. We found that PERSI-WM had larger effect sizes and AUC values between diagnostic groups based on tau retention in specific ROIs. These results demonstrate that PERSI-WM has better diagnostic performance for dementia than the traditional method of using the cerebellum as the reference region.

We used the PERSI-WM method for count normalization in flortaucipir and [^18^F]-APN-1607 imaging. The results obtained for the two tau tracers were consistent; in both cases, the ability to distinguish between diagnostic groups was greater with SUVRs based on WM than with those based on the cerebellum. Additionally, in the anterior cingulate, SUVRs derived from the cerebellum showed high tau retention in MCI and AD patients whereas those derived from PERSI-WM showed higher tau retention in AD patients than in MCI patients. This suggests that in clinical practice, suboptimal count normalization can result in misdiagnosis or inaccurate assessment of disease severity. PERSI has been shown to improve diagnostic accuracy (Southekal et al., 2018). Our results provide evidence that the PERSI-WM method is superior to the conventional method of using the cerebellum as a reference region for the diagnosis of dementia, and suggest that the former is more useful for tau and Aβ PET imaging.

In this study, tau retention was detected in brain regions that are susceptible to neurodegeneration in AD including the frontal, parietal, temporal, and occipital lobes; anterior and posterior cingulate; precuneus; and Braak I/II (entorhinal cortex and hippocampus). Several tau tracers show elevated signals in the temporal region and more broadly throughout the cortex in AD patients than in HCs (Wong et al., 2018; Lohith et al., 2019; Lu et al., 2020; Mueller et al., 2020). Tau retention was detected in Braak I/II in the early stages of AD and MCI (Maass et al., 2017), which is supported by our results. We observed relatively high tau retention in the temporal and occipital lobes in Cohort I (flortaucipir cohort) and in the precuneus and posterior cingulate in Cohort II ([^18^F]-APN-1607); the latter is relatively consistent with a previous report on [^18^F]-APN-1607 tau retention based on effect sizes in the same brain regions, which also found that PERSI-WM had better diagnostic performance than the cerebellar cortex (Lu et al., 2020).

We used the same PERSI method to calculate SUVRs for two tracers in two different cohorts. PERSI-WM could effectively distinguish AD patients from HCs in Cohort II. Based on MMSE and CDR-SB scores, cognitive decline was more severe in AD patients of Cohort II than in those of Cohort I; this suggests that the former had higher tau retention, which could result in a larger effect size and AUC, although further investigation in a larger population is required to confirm this possibility.

In both Cohort I and II, SUVR in HC were <1.0 in some brain regions such as the frontal and parietal lobes, especially with the PERSI method. SUVR of cortex <1.0 have been obtained for other tau tracers using cerebellum GM as a reference region, including [^18^F]-AV-1451 (Shcherbinin et al., 2016), [^18^F]-PI-2620 (Mueller et al., 2020), [^18^F]-MK-6240 (Lohith et al., 2019), and [^18^F]-RO-948 (Wong et al., 2018). Shcherbinin et al. (2016) reported a [^18^F]-AV-1451 study with four young cognitively normal (YCN) subjects, five old cognitively normal (OCN) subjects, five MCI and four AD. Although mean SUVR from 80 to 100 min after injection of all 4 groups were above 1.0 in five target ROIs including the frontal, lateral parietal, occipital, mesial temporal and lateral temporal lobe. However in the time courses of regional SUVR for subjects in each diagnostic category figure, some YCN and/or OCN subjects showed SUVRs <1.0 in the above six target ROIs. Mueller et al. (2020) reported a [^18^F]-PI-2620 study with 10 HC and 12 AD. In the Supplementary Table 1 of Muller’s study, HC showed mean SUVR <1.0 in posterior cingulate from 60 to 90 min after injection. Interestingly, SUVR in subcortical WM was <1.0 (0.90 ± 0.10) at same time. This suggested that PERSI based on WM may have less binding than cerebellum. Lohith et al. (2019) reported a [^18^F]-MK-6240 study with four HC and six MCI or AD. In the regional (representative cortical and subcortical) SUVR-time course with cerebellar cortex as reference figure, 2 HC showed SUVR <1.0 in temporal cortex and hippocampus from 60 to 90 min after injection. Wong et al. (2018) reported a [^18^F]-RO-948 study with six HC and four AD. Supplementary Table 4 of Wong’s study showed SUVR of HC <1 in hippocampus, parahippocampus and insula. Therefore, SUVR of some cortex region <1 maybe reflect non-specific binding of the tracer to neuro-pigments, which has been observed in *in vitro* autoradiography studies. SUVRs <1 can mostly be attributed to off-target binding in WM (Lohith et al., 2019). The WM is a key aspect of the PERSI method that can yield lower SUVRs compared to the cerebellum.

This study had several shortcomings. (1) The size of the [^18^F]-APN-1607 cohort was relatively small, and only HCs and AD patients were included. The PERSI method is a data-driven approach for reducing spatial variability; therefore, additional [^18^F]-APN-1607 data are needed to fully verify the applicability of the PERSI method for the diagnosis of dementia, including from different disease stages such as MCI. (2) In a [^18^F]-APN-1607 PET study, AD patients showed higher binding than HCs in regions outside the cerebral cortex such as the caudate and putamen (Lu et al., 2020); and in a flortaucipir study, the putamen showed higher binding in AD patients than in HCs. We focused only on ROIs in the cerebral cortex in the present study, but future investigations should include more ROIs in other brain regions. (3) For longitudinal studies, the PERSI method can mitigate the influence of the PVE. AD is a chronic neurodegenerative disease with long course, with AD patients followed up multiple times over a long period. As both cortical atrophy and tracer uptake kinetics can change over time, the WM as a reference region can be variably affected at different time points. PERSI can alleviate the PVE by detecting the optimal reference signal based on voxel intensity, such that the results are less affected by differences in uptake pattern or image processing errors at different time points (Landau et al., 2015; Southekal et al., 2018). There were no longitudinal data for the two tau tracers examined in this study, although these could enhance the applicability of the PERSI method. (4) In the present study, the flortaucipir data were obtained from the ADNI database; as such, our analyses were retrospective. A prospective study with flortaucipir PET is needed to validate our results. (5) Although we tested the PERSI method with different tracers in two cohorts, the study population included only AD patients with amyloid deposition, with no amyloid-negative dementia patients. For more definitive conclusions, amyloid status and findings from anatomic MRI or glucose metabolism PET imaging should be considered. (6) Because of the small size of Cohort II ([^18^F]-APN-1607 PET), we did not examine voxel-wise correlations between cognitive impairment (e.g., MMSE score) and [^18^F]-APN-1607 binding in the cerebral cortex of AD patients, although this is important to confirm the broader clinical utility of the PERSI method.

## Conclusion

The PERSI-WM method is superior to the conventional method of using the cerebellum as a reference region for semi-quantification of tau deposition and detection of multiple tau tracers in PET imaging; it can also mitigate the influence of the PVE. Thus, the PERSI method can be promising for the accurate diagnosis of dementia based on PET imaging.

## Alzheimer’s Disease Neuroimaging Initiative

Alzheimer’s Association; Alzheimer’s Drug Discovery Foundation; Araclon Biotech; BioClinica, Inc.; Biogen; Bristol-Myers Squibb Company; CereSpir, Inc.; Eisai, Inc.; Elan Pharmaceuticals, Inc.; Eli Lilly and Company; EuroImmun; F. Hoffmann-La Roche Ltd. and its affiliated company Genentech, Inc.; Fujirebio; GE Healthcare; IXICO, Ltd.; Janssen Alzheimer Immunotherapy Research & Development, LLC; Johnson & Johnson Pharmaceutical Research & Development LLC; Lumosity; Lundbeck; Merck & Co., Inc.; Meso Scale Diagnostics, LLC.; NeuroRx Research; Neurotrack Technologies; Novartis Pharmaceuticals Corporation; Pfizer, Inc.; Piramal Imaging; Servier; Takeda Pharmaceutical Company; and Transition Therapeutics. The Canadian Institutes of Health Research is providing funds to support ADNI clinical sites in Canada. Private sector contributions are facilitated by the Foundation for the National Institutes of Health (https://www.fnih.org). The grantee organization was the Northern California Institute for Research and Education, and the study was coordinated by the Alzheimer’s Disease Cooperative Study at the University of California, San Diego. ADNI data are disseminated by the Laboratory for Neuroimaging at the University of Southern California.

## Data Availability Statement

The raw data supporting the conclusions of this article will be made available by the authors, without undue reservation.

## Ethics Statement

The studies involving human participants were reviewed and approved by the Institutional Review Board of Huashan Hospital (HIRB), Fudan University. The patients/participants provided their written informed consent to participate in this study.

## Author Contributions

HZ and MW conceived and designed the experiments, analyzed and interpreted the data, and wrote the manuscript. JL, WB, and LL performed the experiments and wrote the manuscript. CZ and JJ analyzed and interpreted the data and wrote the manuscript. All authors read and approved the final version of the article for publication.

## Conflict of Interest

The authors declare that the research was conducted in the absence of any commercial or financial relationships that could be construed as a potential conflict of interest.

## Footnotes

1 adni.loni.usc.edu

2 http://adni.loni.usc.edu/methods/documents/
